# Cost effectiveness analysis of single and sequential testing strategies for tuberculosis infection in adults living with HIV in the United States

**DOI:** 10.1038/s41598-022-22721-z

**Published:** 2022-11-01

**Authors:** Ginenus Fekadu, Jiaqi Yao, Joyce H. S. You

**Affiliations:** Faculty of Medicine, School of Pharmacy, The Chinese University of Hong Kong, Hong Kong SAR, China

**Keywords:** Tuberculosis, Diagnosis, Disease prevention, Health care economics, Public health

## Abstract

Tuberculosis infection (TBI) frequently progresses to tuberculosis (TB) disease in people co-infected with human immunodeficiency virus (HIV). We examined the cost-effectiveness of single, sequential and no testing (total 12) strategies of TBI in HIV-infected people from the perspective of US healthcare provider. A decision-analytic model (20-year timeframe) was constructed to simulate TB-related outcomes: Direct medical cost and quality-adjusted life-years (QALYs). In the base-case analysis, the “confirm negative TST followed by QFT-Plus” strategy gained 0.1170 QALY at a total cost of USD3377. In the probabilistic sensitivity analysis of 10,000 Monte Carlo simulations, the probability of “confirm negative TST followed by QFT-Plus” to be accepted as cost-effective was the highest of all 12 strategies when the willingness-to-pay threshold exceeded 2340 USD/QALY. In conclusion, the strategy of “confirm negative TST followed by QFT-Plus” appears to be the preferred cost-effective option for TBI testing in HIV-infected people from the US healthcare provider’s perspective.

## Introduction

About a quarter of the world’s population is estimated to be infected with *Microbacterium tuberculosis*, including approximately 13 million people in the United States (US)^1–3^. The majority of the patients remain in a state of tuberculosis infection (TBI) without symptoms of tuberculosis (TB) disease. About 5–10% of those with TBI progress to TB disease in their lifetime, and co-infection with human immunodeficiency virus (HIV) is associated with a 20-fold increase in the risk of TBI-to-TB progression^2,4^. TBI has been recognized as the largest source of new TB cases globally, and more than 80% of TB cases in the US resulted from longstanding, untreated TBI^5,6^. In the US, the societal benefits of preventive treatment of TB ranged from USD6.7 to USD14.5 billion during 1995–2014^7^.

TB is the leading cause of acquired immunodeficiency syndrome (AIDS)-related death globally. Over 214,000 TB fatalities were reported in 2020, accounting for roughly one-third of all deaths in HIV-infected people^8^. The US prevalence of TBI among HIV-infected patients was 7.6% (3.3–16.7%)^9^, with a higher prevalence in those who were foreign-born (31.7%) versus US-born (4.20%)^10^. Identifying and treating TBI is a critical component to prevent TB in people infected with HIV^2,5^. A systematic review of 12 randomized controlled trials in HIV-infected people found that treatment of TBI reduced the overall TB risk by 33%^11^. The early treatment of TBI also significantly reduced the risk of TB mortality by 26% in people infected with HIV^12^.

The detection of TBI mainly depends on the host’s immune response to the antigens of *Microbacterium tuberculosis*^13^. Tuberculin skin test (TST), a purified protein derivative skin test, has been widely used to detect TBI for over 100 years^13,14^. The TST is convenient (with no laboratory requirements) and inexpensive, yet limited by low specificity, which is caused by cross-reactivity with Bacille Calmette-Guerin (BCG) vaccine and non-tuberculosis mycobacteria (NTM)^15,16^. Interferon-gamma release assays (IGRAs) are in vitro blood tests that quantify the cellular immune response by detecting IFN-γ release in response to antigens ESAT-6 and CFP-10 stimulation^15,16^. The IGRAs offer some advantages over the TST, including higher specificity, and less cross-reactivity with NTM and BCG vaccines. The IGRAs require sophisticated laboratory infrastructure and therefore are relatively more costly than TST^13,17^. The currently available IGRAs approved by the Food and Drug Administration (FDA) in the US are the T-cell spot of the TB assay (T-SPOT.TB) and the QuantiFERON-TB Gold Plus (QFT-Plus)^18^.

The sensitivities of TST and IGRA tests are reduced in immunocompromised patients, and therefore increase the risk of false-negative results^17,19^. The strategies of sequential testing might be useful to improve TBI testing accuracy by increasing sensitivity and have been suggested for individuals who are likely to be infected and at high risk of progression (including people infected with HIV)^5,20^. To inform the health policy and service provider on the outcomes of different testing strategies for TBI, this study aimed to examine the clinical and health economic outcomes of 12 testing strategies, including single and sequential testing, of TBI in HIV-infected people from the perspective of healthcare provider in the US.

## Methods

### Model design

**Figure 1:**
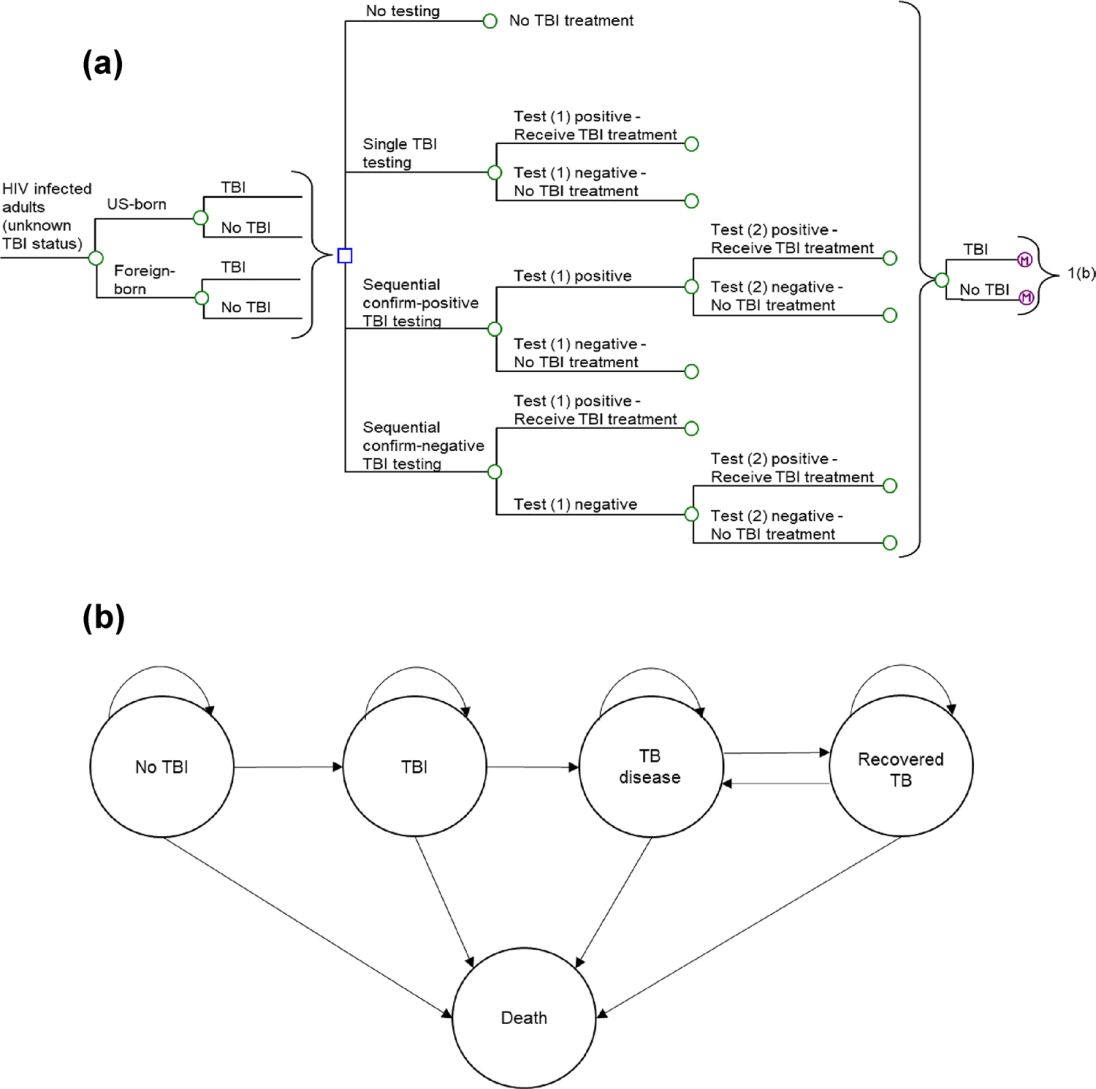
Simplified decision-analytic model. 12 testing strategies are: no testing approach, 3 single TBI testing approaches (TST; QFT-Plus; T-SPOT.TB), 4 sequential confirm-positive TBI testing approaches (confirm positive TST followed by QFT-Plus; confirm positive TST followed by T-SPOT.TB; confirm positive QFT-Plus followed by TST; confirm positive T-SPOT.TB followed by TST), and 4 sequential confirm-negative TBI testing approaches (confirm negative TST followed by QFT-Plus; confirm negative QFT-Plus followed by TST; confirm negative TST followed by T-SPOT.TB; confirm negative T-SPOT.TB followed by TST). HIV: human immunodeficiency virus; TBI: tuberculosis infection; TB: tuberculosis.

A decision-analytic model, including a short-term decision tree model followed by a 20-year Markov model, was constructed to simulate the long-term TB-related outcomes of a hypothetical cohort of adult HIV-infected individuals undergoing TBI screening from the perspective of healthcare provider in the US (Fig. 1a,b).

Decision-analytic modelling is a well-accepted tool to provide a computational framework for evaluation of the cost and clinical outcomes of health interventions over time, using evidence-based probabilities of clinical events, utility and cost inputs from multiple sources^21,22^. Model-based analysis examines the result robustness over the model input uncertainties using sensitivity analysis, providing information on thresholds of influential parameters on the cost-effectiveness of the health intervention^23^. The cost-effectiveness findings generated by decision-analytic modelling facilitate policy and decision makers on informed decision of resources allocation to generate optimal benefit on population health^24^.

All hypothetical individuals (both US-born and foreign-born) first entered a decision tree model to evaluate the immediate outcomes of TBI testing and subsequent treatment (Fig. 1a). A total of twelve testing strategies were examined: No testing strategy, three single-test strategies (TST; T-SPOT.TB; QFT-Plus), four sequential strategies to confirm negative tests (TST-negative followed by T-SPOT.TB; TST-negative followed by QFT-Plus; T-SPOT.TB-negative followed by TST and QFT-Plus-negative followed by TST) and four sequential strategies to confirm positive tests (TST-positive followed by T-SPOT.TB, TST-positive followed by QFT-Plus, T-SPOT.TB-positive followed by TST and QFT-Plus-positive followed by TST).

In all testing strategies, a hypothetical individual might or might not have TBI, and this individual might be either not tested, or tested positive or negative for TBI. For no testing strategy, no TBI testing was carried out and no TBI treatment was provided. For the three single-test strategies, a test-positive result indicated for treatment of TBI and a test-negative result required no sequential testing or treatment. In the four confirm-negative sequential strategies, an initial positive testing indicated for treatment. An initial negative testing required a sequential testing (as specified in the strategy), and treatment was provided to those with a positive result. In the four confirm-positive sequential strategies, a sequential testing was conducted for those with initial positive testing, and treatment was indicated for those who were tested positive in both sequential tests. After the testings, the individual (with or without TBI) might or might receive treatment of TBI (3-month once-weekly rifapentine 900 mg plus isoniazid 900 mg (3HP)^6,14^) and further entered the Markov model (at the health state of “TBI” or “no TBI”) for a maximum of 20 years with yearly cycle.

The Markov model resembled the disease progression of TBI, including five health states: “no TBI”, “TBI”, “TB disease”, “TB recovered”, and “death” (Fig. 1b). The hypothetical individuals proceeded through health states in each model cycle according to transition probabilities. All individuals might die from all causes in every yearly cycle. Of those who survive in each yearly cycle, the individuals with no TBI might acquire TBI. Patients in the “TBI” state might develop TB disease. TB disease patients were managed according to the CDC guidelines for adults with HIV^14^, and the patients might or might not achieve treatment success. Patients proceeded to the “TB recovered” state if the treatment success was achieved, and the recovered patients might experience TB disease recurrence in the following yearly cycles. Those with TB disease who failed treatment would die or receive palliative care until death.

### Clinical inputs

All model inputs are shown in Table 1. A search for clinical probabilities was performed in the Medline covering the period 2000–2022 and public data of the CDC and World Health Organization (WHO) to estimate input parameters for the model using keywords “Mycobacterium infection”; “latent tuberculosis infection”; “active tuberculosis”; “tuberculosis skin test”; “interferon-gamma release assay”; “T-SPOT.TB”; “QFT-Plus”; and “human immunodeficiency virus infection”. The inclusion criteria for clinical studies were: (1) Reports written in English; (2) adult HIV-infected patients and (3) TBI test results and/or treatment outcomes were reported. Systematic review and meta-analysis were preferred sources for the model inputs. A study was included if the data relevant to the model inputs were available. If multiple sources were obtained for model input, the weighted average was used as the base-case value, and the high or low values formed as the range for sensitivity analysis.

**Table 1:** Model input parameters.

Parameters	Base-case value	Range for sensitivity analysis	Distribution	Reference
**Clinical inputs**				
Proportion of foreign-born among HIV patients	18.90%	15.12–22.68%	Beta	^25^
TBI prevalence in foreign-born HIV-infected patients	31.70%	1.90–46.80%	Beta	^10^
TBI prevalence in US-born HIV-infected patients	4.20%	2.60–6.30%	Beta	^10^
Sensitivity (foreign-born)				^10^
TST	69.10%	58.50–79.70%	Beta	
T-SPOT.TB	58.90%	42.70–76.20%	Beta	
QFT-Plus	71.20%	55.30–86.50%	Beta	
Sensitivity (US-born)				^10^
TST	54.00%	44.20–64.30%	Beta	
T-SPOT.TB	55.00%	40.70–70.60%	Beta	
QFT-Plus	67.50%	52.90–81.70%	Beta	
Specificity (foreign-born)				^10^
TST	88.50%	70.70–96.00%	Beta	
T-SPOT.TB	97.20%	84.10–99.70%	Beta	
QFT-Plus	93.10%	73.90–99.10%	Beta	
Specificity (US-born)				^10^
TST	96.50%	95.30–97.60%	Beta	
T-SPOT.TB	99.30%	98.60–99.80%	Beta	
QFT-Plus	95.80%	94.40–97.10%	Beta	
Treatment completion rate of TBI treatment	85.40%	80.40–89.40%	Beta	^26^
TB risk reduction rate after completed TBI treatment	90.00%	75.00–100.00%	Beta	^27,28^
The annual rate of TB reactivation in HIV-infected patients	1.82%	1.74–1.89%	Beta	^29^
Treatment success rate of HIV-positive TB disease	70.00%	56.00–84.00%	Beta	^30^
Mortality rate among not successfully treated TB-HIV patients	59.65%	47.72–71.58%	Beta	^30^
Annual TB recurrence rate in HIV-infected patients	4.09%	2.31–6.65%	Beta	^31^
**Utility inputs**				
TBI	0.92	–		^10,36^
TB disease	0.69	0.57–0.77	Uniform	^37^
TB disease treatment success	0.88	0.77–1.00	Uniform	^37,38^
HIV patient age (years)				^10^
Foreign-born	46.4	37.6–56.0	Triangular	
US-born	49.6	42.6–54.8	Triangular	
**Cost inputs (USD)**				
Cost per test				
TST	35	28–42	Gamma	^39^
T-SPOT.TB	100	80–120	Gamma	^40^
QFT-Plus	62	50–74	Gamma	^40^
Cost per case				
TBI	710	568–852	Gamma	^41^
TB disease	21,955	17,564–26,346	Gamma	^42^
Palliative care	27,158	21,726–32,590	Gamma	^43^
TB-related mortality	37,499	29,999–44,999	Gamma	^42^

*HIV* human immunodeficiency virus, *TBI* tuberculosis infection, *QFT-Plus* QuantiFERON-TB Gold Plus, *T-SPOT.TB* T-cell spot of the TB assay, *TST* tuberculin skin test, *TB* tuberculosis.

The proportion of foreign-born (18.9%) and US-born (81.1%) individuals among HIV-infected patients were approximated from the findings of a cross-sectional study on epidemiology data from the National HIV Surveillance System for people with HIV infection (n = 328,317 patients) during 2010–2017^25^. A prospective clinical study (n = 10,740) on TBI diagnostics reported the TBI prevalence among subgroups based on age, foreign birth outside the USA, and HIV infection. The prevalence of TBI was reported to be 31.7% in foreign-born and 4.20% in US-born HIV-infected patients^10^. The study also reported the sensitivities and specificities of TBI testing strategies both in foreign-born and US-born HIV-infected patients, and were adopted for the model inputs of sensitivities and specificities of TST, T-SPOT.TB and QFT-Plus^10^.

The treatment of TBI (3HP once-weekly) completion rate was approximated from the results of the directly observed therapy for TBI in an open-label, phase 4 randomized clinical trial (n = 1002 patients) in the outpatient tuberculosis clinics in the US, Spain, Hong Kong, and South Africa. Of those patients enrolled in the US, the treatment completion rate was 85.40%^26^. A prior US health economic analysis of TBI screening for at-risk populations had adopted a 90% TB risk reduction from the 9-month isoniazid preventive treatment^27^, and a randomized noninferiority trial (n = 7731) found 3HP therapy to be as effective as the 9-months isoniazid therapy in subjects at high-risk for TB^28^. The present model, therefore, adopted 90% as the TB risk reduction from the 3HP treatment for TBI. The outcomes of TBI patients who initiated but failed to complete the TBI treatment were assumed to be the same as those without treatment.

The yearly probability of progression from TBI to TB disease in HIV-infected patients (1.82%) was approximated from the results of an epidemiology study of reactivation TB in the US, using data on TB cases (n = 39,920) reported to the CDC during 2006–2008^29^. The WHO reported the treatment success rate of TB disease among HIV-infected patients to be 70.00% in the US, and the mortality rate among those who failed anti-TB therapy was estimated to be 59.65%^30^. The yearly TB recurrence probability among HIV-infected patients (4.09%) was estimated from the findings of two prospective clinical trials conducted by the Tuberculosis Trials Consortium in the US and Canada (n = 1244 culture-positive TB patients) on the outcomes of anti-TB treatment^31^. Age-specific all-cause mortality rates were retrieved from the US Life table reported by WHO^32^.

### Health utility inputs

The effectiveness of testing strategies was evaluated in terms of the quality-adjusted life-year (QALY), estimated using the utility and duration of time spent in each of the health states^33^. The health utility weights of TBI were not significantly different from those of the healthy general population^34,35^. Hence, the utility model input of TBI adopted the age-specific health utility (0.92 for age 18–65 years), previously generated by the -related quality of life study using the US national health measures and surveys^36^. The base-case age of the HIV-infected individuals for foreign-born (46.4 years) and US-born (49.6 years) were retrieved from the prospective clinical study evaluating TBI diagnostics in the US^10^. The utility value of TB disease (0.69) was approximated from a cross-sectional survey on the impact of TB on health utility^37^. The utility of TB disease treatment success (0.88) was retrieved from the findings of health-related quality of life studies in TB patients^37,38^. The QALYs accumulated over the model time horizon were discounted to the year 2022, with an annual discounting rate of 3%.

### Cost inputs

The cost analysis was conducted from the perspective of the US healthcare provider. Direct medical costs included in the model were costs of diagnostic tests, treatment of TBI, TB disease treatment, palliative care, and TB-related mortality. The cost of TST was approximated from the Medicare-allowable physician fee schedule^39^. Cost of T-SPOT.TB and QFT-Plus were adopted from Centres for Medicare and Medicaid Services clinical laboratory fee schedule^40^. The direct treatment cost per case of TBI was obtained from published reports by CDC^41^. Similarly, according to a CDC report, the per-patient cost of treating TB was USD21,955, and the cost of TB-related mortality was USD37,499^42^. The direct cost of palliative care per admission was retrieved from a meta-analysis study of the health economics of palliative care for hospitalized adults with serious illness^43^. All costs in the present analysis were adjusted to the year 2022, according to the Consumer Price Index in the US^44^. The future incurred costs were discounted to the year 2022 by 3% annually.

### Cost-effectiveness, sensitivity and scenario analyses

All analyses were performed using TreeAge Pro 2022 (TreeAge Software Inc, Williamstown, MA, USA) and Excel 365 (Microsoft Corporation, Redmond, WA, USA). The primary outcomes of the model were direct medical cost, QALY, and incremental cost per QALY gained (ICER). The ICER of a strategy was calculated if the strategy gained additional QALYs at a higher cost than the next less costly strategy: ICER = ∆Cost/∆QALYs.

A strategy was dominated when it gained either (1) lower QALYs at a higher cost or (2) lower QALYs with higher ICER than another strategy. The dominated strategies were removed from further cost-effectiveness analysis. A strategy was accepted as the preferred cost-effective treatment if it gained (1) higher QALYs at a lower cost, or (2) higher QALYs at a higher cost, and the ICER was less than the willingness-to-pay (WTP) threshold^22,45^.

All model inputs were examined by the one-way sensitivity analysis over the ranges specified in Table 1. The one-way sensitivity analysis was used to describe the association between each input variable and the primary outcomes (cost and QALY) for examination of the robustness of base-case findings. The cost and QALY were further recalculated 10,000 times in the probabilistic sensitivity analysis using Monte Carlo simulations, by randomly drawing each model input value from the parameter-specific distribution, to evaluate the impact of uncertainty in all variables simultaneously. The details of distributions used in the probabilistic sensitivity analysis are provided in Supplementary Table S1. The mean incremental cost and QALY gained along with the 2.5th and 97.5th percentiles were computed to estimate the uncertainty interval of the simulation. The probabilities of each strategy to be accepted as cost-effective were assessed in the acceptability curves over a broad range of WTP threshold from 0 to 200,000 USD/QALY^46–48^.

In the present study, all individuals were tested for TBI once at the entry of the model. Individuals who are at high risk for ongoing or repeated exposure to TB disease, such as incarceration, traveling in a high-TB incidence area, homelessness, and living in a congregate setting, are recommended to test for TBI yearly^5,14^. A scenario analysis on yearly TBI screening with the same 12 testing strategies was conducted for HIV-infected individuals who were at high risk for exposure to TB disease.

## Results

### Base-case analysis

**Figure 2:**
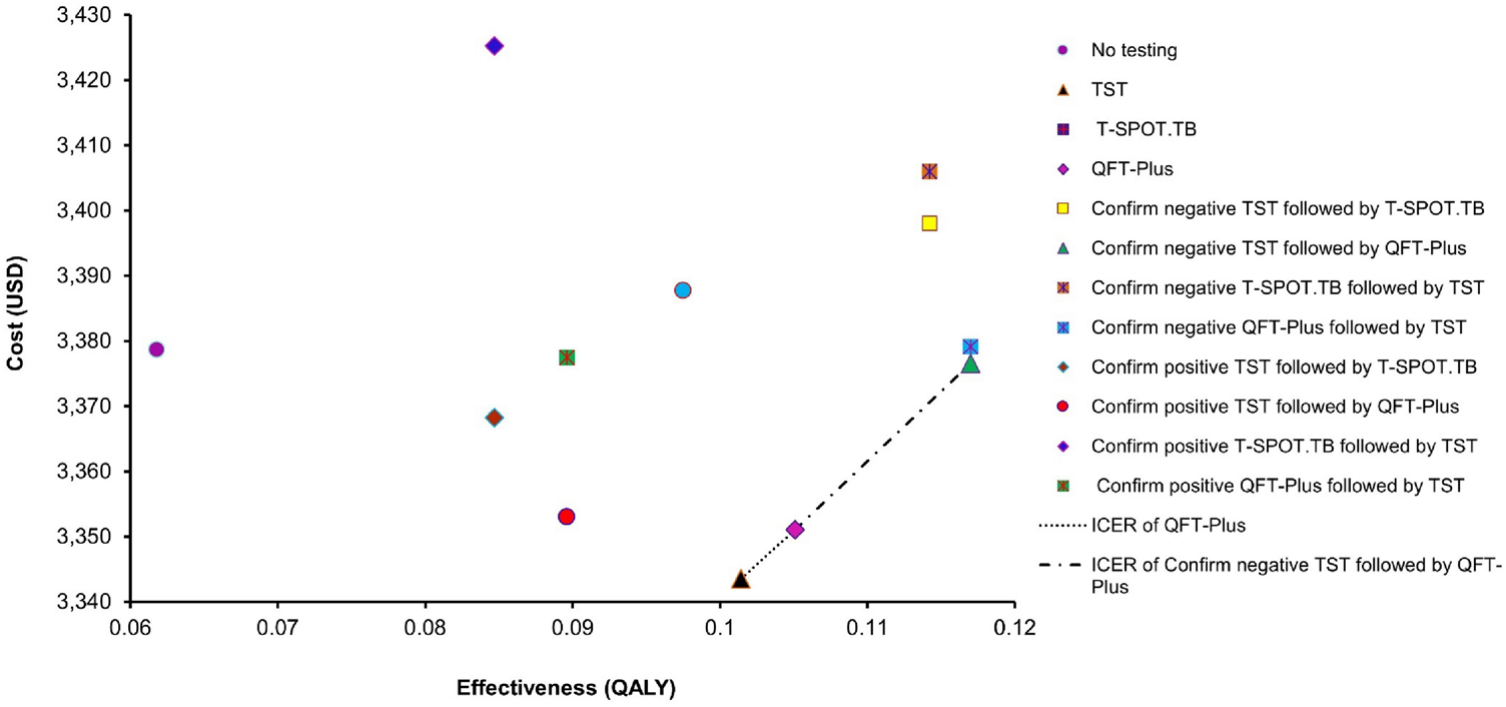
Base-case cost-effectiveness plot (cost versus QALY) of 12 strategies for TBI testing. *QALY* quality-adjusted life years, *ICER* incremental cost-effectiveness ratio, *TBI* tuberculosis infection, *QFT-Plus* QuantiFERON-TB Gold Plus, *T-SPOT.TB* T-cell spot of the TB assay, *TST* tuberculin skin test.

The base-case expected cost versus expected QALY gained of all testing strategies are shown in Fig. 2. Of the 12 strategies examined, 9 strategies were dominated and eliminated from further cost-effectiveness analysis. Three testing strategies remained in the cost-effectiveness analysis: (1) TST, (2) QFT-Plus, and (3) confirm negative TST followed by QFT-Plus. The base-case results of non-dominated strategies are shown Table 2.

**Table 2:** Base-case results of un-dominated strategies.

Un-dominated strategies	Total direct cost (USD)	Incremental cost (vs. the next less costly option)	QALYs	QALY gained (vs. the next less costly option)	ICER (USD/QALY)
TST	3344	–	0.1014	–	–
QFT-Plus	3351	7	0.1051	0.0037	1892
Confirm negative TST followed by QFT-Plus	3377	26	0.1170	0.0119	2185

*ICER* = incremental cost/QALY gained.

*QALY* quality-adjusted life years, *ICER* incremental cost-effectiveness ratio, *QFT-Plus* QuantiFERON-TB Gold Plus, *TST* tuberculin skin test.

Comparing with the TST strategy, the “QFT-Plus” strategy gained 0.0037 QALYs at a higher cost of USD7 (ICER = 1892 USD/QALY gained). When compared to the QFT-Plus, the “confirm negative TST followed by QFT-Plus” gained 0.0119 QALYs with an incremental cost of USD26 (ICER = 2185 USD/QALY gained). The base-case results of all 12 testing strategies are shown in Supplementary Table S2.

### One-way sensitivity analysis

The base-case results were robust to the variation of all model inputs in the one-way sensitivity analysis, and no threshold parameter was identified to change the base-case cost-effectiveness results. A tornado diagram (Supplementary Fig. S1**)** shown the top five influential parameters on the “confirm negative TST followed by QFT-Plus” strategy: TBI prevalence in foreign-born HIV patients, TB disease treatment success rate, TBI prevalence in US-born HIV patients, proportion of foreign-born among HIV patients, and TB risk reduction rate after completed TBI treatment.

### Probabilistic sensitivity analysis

**Figure 3:**
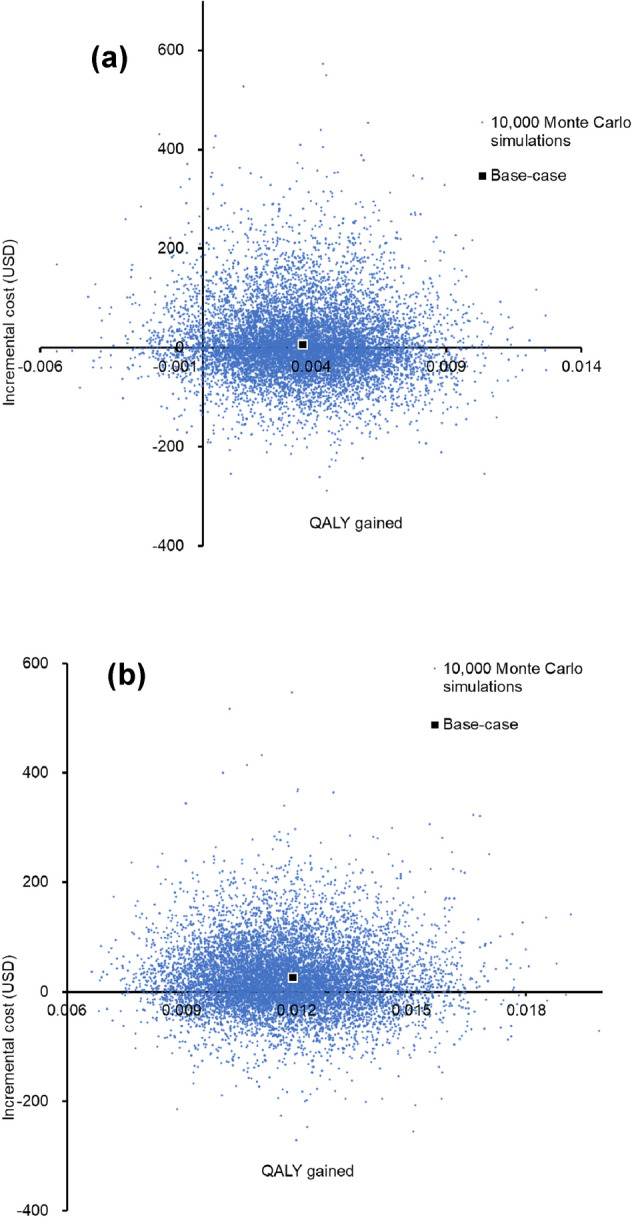
Scatter plot of the incremental cost against QALY gained with (**a**) “QFT-Plus” versus “TST”; (**b**) “Confirm negative TST followed by QFT-Plus” versus “QFT-Plus” in 10,000 Monte-Carlo simulations. *QALY* quality-adjusted life-year, *TBI* tuberculosis infection, *QFT-Plus* QuantiFERON-TB Gold Plus, *TST* tuberculin skin test.

Probabilistic sensitivity analysis was conducted by recalculating the cost and QALYs 10,000 times with Monte Carlo simulation. Compared with the TST strategy, the “QFT-Plus” strategy showed a mean QALY increment of 0.0037 (2.5th percentile: − 0.0008; 97.5th percentile: 0.0083), and a mean incremental cost of USD10 (2.5th percentile: − USD118; 97.5th percentile: USD189). The incremental cost versus additional QALY gained by the “QFT-Plus” strategy was shown in Fig. 3a.

When compared with the “QFT-Plus” strategy, the “confirm negative TST followed by QFT-Plus” strategy was more costly by a mean incremental cost of USD25 (2.5th percentile: − USD85; 97.5th percentile: USD169) and gained 0.0117 QALYs (2.5th percentile: 0.0086; 97.5th percentile: 0.0154). The incremental cost versus additional QALY gained by the “confirm negative TST followed by QFT-Plus” strategy was shown in Fig. 3b.

**Figure 4:**
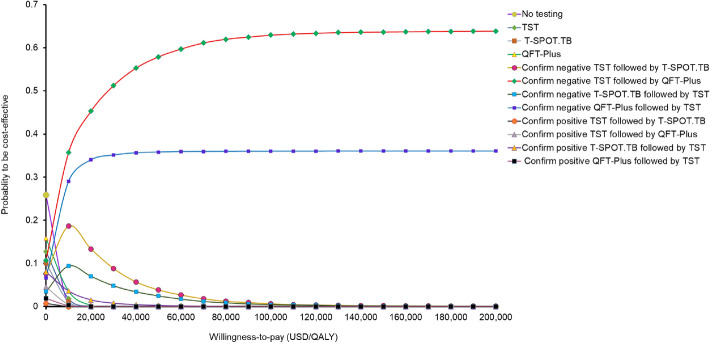
Acceptability curves of 12 strategies for TBI testing to be cost-effective against willingness-to-pay. *TBI* tuberculosis infection, *QALY* quality-adjusted life years, *QFT-Plus* QuantiFERON-TB Gold Plus, *T-SPOT.TB* T-cell spot of the TB assay, *TST* tuberculin skin test, *WTP* willingness-to-pay.

The probability of each strategy to be cost-effective against the WTP threshold (over the range of 0–200,000USD/QALY) was presented in the cost-effectiveness acceptability curves (Fig. 4). The probability of “confirm negative TST followed by QFT-Plus” to be accepted as cost-effective was the highest of all 12 strategies when the WTP threshold exceeded 2340 USD/QALY. At the WTP thresholds of 50,000, 100,000 and 150,000 USD/QALY, the probabilities of “confirm negative TST followed by QFT-Plus” to be accepted as cost-effective were 57.89, 62.93 and 63.65%, respectively.

### Scenario analysis

When yearly TBI testing was applied in the present model, the “confirm negative TST followed by QFT-Plus” strategy showed an ICER of 7429 USD/QALY. The probabilistic sensitivity analysis of 10,000 Monte Carlo simulations found that the “confirm negative TST followed by QFT-Plus” was the preferred strategy with the highest probability (45.48, 54.97 and 58.94%) to be cost-effective at the WTP threshold of 50,000, 100,000 and 150,000 USD/QALY, respectively.

## Discussion

This is the first cost-effectiveness analysis to compare multiple TBI testing (including single, sequential, and no testing) strategies for both US-born and foreign-born HIV-infected adults in a low TB burden and high-income setting. The findings showed that the “confirm negative TST followed by QFT-Plus” yielded the greatest benefits from the perspective of US healthcare provider over the time horizon of 20 years. In the probabilistic 10,000 Monte Carlo simulations, the probability of “confirm negative TST followed by QFT-Plus” to be accepted as cost-effective was the highest of all 12 strategies when the willingness-to-pay threshold exceeded 2340 USD/QALY.

A UK study examined five TBI testing strategies for HIV-infected individual. The QuantiFERON-TB Gold In-Tube negative followed by TST strategy was the most costly and effective (ICER = £20,163/QALY), with a probability of 41% to be cost-effective at thresholds above £40,000 per QALY^49^. Another model-based cost-effectiveness study of testing for TBI also found that confirm sequential negative test (IGRA followed by TST) in non-US-born HIV patients was the preferred cost-effective strategy, with an incremental QALY (0.0008) at incremental cost USD 50 per person and ICER 63,000 USD/QALY (compared to no testing)^48^. The present findings were similar to those reported by these two studies that confirm negative test was consistently the preferred cost-effective strategy.

The main driving reason for the preference of the “confirm negative TST followed by QFT-Plus” strategy was the high accuracy to identify a TBI case due to relative higher sensitivity of QFT-Plus. Accurate identification of TBI at testing led to the timely treatment of TBI. Our findings showed that there is a cost-effectiveness benefit of TBI testing when compared to “no testing” for HIV-infected individuals. The risk for progression to TB disease is increased when the TBI is untreated in patients living with HIV^2^, thus further worsens the TB transmission and TB-related morbidity and mortality. TB-related morbidity and mortality were associated with high direct medical costs (USD21,000–38,000)^42^.

Confirm positive testing strategies were associated with lower QALYs. The unfavourable outcomes were driven by the overall increased specificities and yet compromised sensitivity of strategies. The risk of the TBI patients to have false-negative testing results and be undiagnosed was therefore elevated, resulting in higher risk of progression to TB disease and higher TB-related disablity and mortality.

Individuals who were at high risk for ongoing or repeat exposure to persons with TB disease are recommended to screen for TBI annually^5,14^. The scenario analysis examined annual TBI testing and found that “confirm negative TST followed by QFT-Plus” was the preferred cost-effective option. The “confirm negative TST followed by QFT-Plus” was preferred option because of higher sensitivity of QFT-Plus^10^. Further study on yearly TBI testing for high-risk individual is highly warranted to analyse the driving factors of a cost-effective TBI testing strategy.

According to current CDC recommendations, IGRA or TST can be used for testing TBI. Sequential testing is also recommended by CDC for persons who are likely to be infected and have a high risk of progression such as patients living with HIV^5,20^. The present study has some important implications for addressing TB elimination among HIV-infected individuals. Our findings supported that, for HIV-infected individual in whom both the prevalence of TBI and the risk of reactivation are high, testing algorithms should maximize test sensitivity. When two tests are applied sequentially, the overall sensitivity is higher than either test alone. The serial sequential confirm negative testing increases overall sensitivity, and therefore reduces false negative results and thus fewer undiagnosed cases.

There is no empirical and consensus WTP threshold value in the US^47^. The commonly referenced threshold values (50,000 USD/QALY or 100,000 USD/QALY) are arbitrary standards that have been criticized due to a lack of clear methodological and economic justification^50,51^. These thresholds might therefore be over the willingness-to-pay from the perspective of people with TBI who are at high risk for progression to TB disease (lower socioeconomic status including immigrants, unprivileged, homeless, or living in a congregate setting). Further WTP threshold and cost-effective studies are warranted to evaluate the impact of TBI testing from the perspective of patients with HIV.

The present cost-effectiveness study establishes the framework to inform clinical and administrative decision-makers on TBI testing strategies. The model inputs of sensitivity and specificity provide the TBI testing performance targets for future research work to enhance testing accuracy. Understanding the impact of cost-effectiveness drivers (influential factors) on the decision-making processes facilitates the researchers in fields of diagnostic pathology and clinical chemistry on TBI testing to aim the research at the target sensitivity/specificity. The model framework developed in the present study is also readily to be applied by health policymakers and clinicians in other countries, using the region-specific clinical and economic model inputs, to maximize the health economic outcomes of TBI testing strategies in HIV-infected individuals.

The present study was limited by the inherent model uncertainty. Rigorous sensitivity analyses were performed to examine model uncertainty. The present model focused on a high-income country with low TB prevalence, and the WHO algorithm for people living with HIV in high TB prevalence regions (do not require a TB infection diagnostic test prior to TBI treatment) was therefore not considered. The present findings lacked stratification by TBI prevalence, and the applicability and generalization of the study results are limited to low prevalence countries. Direct medical costs were included in the present model and indirect (such as loss of productivity and transportation) costs were not considered. The cost-effectiveness results might therefore underestimate the health economic benefits generated by preferred strategy for early TBI testing and treatment. Health technology assessment of TBI screening including both direct and indirect costs from a societal perspective is highly warranted.

In conclusion, the strategy “confirm negative TST followed by QFT-Plus” appears to be the preferred cost-effective option for TBI testing in HIV-infected people from the perspective of healthcare provider in the US.

## Supplementary Information

Supplementary Information.

## Data Availability

The authors confirm that the data supporting the findings of this study are available within the article [and/or] its supplementary materials.

## References

[1] Cohen A, Mathiasen VD, Schön T, Wejse C (2019). The global prevalence of latent tuberculosis: A systematic review and meta-analysis. Eur. Respir. J..

[2] World Health Organization. *WHO consolidated guidelines on tuberculosis: module 1: Prevention; Tuberculosis preventive treatment*. https://www.who.int/publications/i/item/9789240001503 (2021). Accessed 8 May 2022.

[3] Centers for Disease Control and Prevention. *Tuberculosis Data and Statistics*. https://www.cdc.gov/tb/statistics/default.htm (2022). Accessed 8 May 2022.

[4] Houben RM, Dodd PJ (2016). The global burden of latent tuberculosis infection: A re-estimation using mathematical modelling. PLoS Med..

[5] Centers for Disease Control and Prevention. *Latent Tuberculosis Infection: A Guide for Primary Health Care Providers*. https://www.cdc.gov/tb/publications/ltbi/pdf/LTBIbooklet508.pdf (2020). Accessed 8 May 2022.

[6] Sterling TR (2020). Guidelines for the treatment of latent tuberculosis infection: Recommendations from the National Tuberculosis Controllers Association and CDC, 2020. Recomm. Rep..

[7] Castro K (2016). Estimating tuberculosis cases and their economic costs averted in the United States over the past two decades. Int. J. Tuberc. Lung Dis..

[8] World Health Organization. *Global Tuberculosis Report 2021*. https://www.who.int/publications/i/item/9789240037021 (2021). Accessed 8 May 2022.

[9] Miramontes R (2015). Tuberculosis infection in the United States: Prevalence estimates from the national health and nutrition examination survey, 2011–2012. PLoS ONE.

[10] Stout JE (2018). Evaluating latent tuberculosis infection diagnostics using latent class analysis. Thorax.

[11] Akolo C, Adetifa I, Shepperd S, Volmink J (2010). Treatment of latent tuberculosis infection in HIV infected persons. Cochrane Database Syst. Rev..

[12] National HIV Curriculum. *Latent Tuberculosis Infection*. https://www.hiv.uw.edu/go/co-occurring-conditions/latent-tuberculosis/core-concept/all (2021). Accessed 8 May 2022.

[13] Gong W, Wu X (2021). Differential diagnosis of latent tuberculosis infection and active tuberculosis: A key to a successful tuberculosis control strategy. Front. Microbiol..

[14] Panel on opportunistic Infections in Adults and Adolescents with HIV. *Guidelines for the prevention and treatment of opportunistic infections in adults and adolescents with HIV: recommendations from the Centers for Disease Control and Prevention*. https://clinicalinfo.hiv.gov/en/guidelines/adult-and-adolescent-opportunistic-infection/mycobacterium-tuberculosis-infection-and?view=full (2021). Accessed 8 May 2022.

[15] Hamada Y (2021). Tests for tuberculosis infection: Landscape analysis. Eur. Respir. J..

[16] Migliori GB (2021). The definition of tuberculosis infection based on the spectrum of tuberculosis disease. Breathe.

[17] Pai M (2014). Gamma interferon release assays for detection of Mycobacterium tuberculosis infection. Clin. Microbiol. Rev..

[18] Centers for Disease Control and Prevention. *Testing for TB Infection*. https://www.cdc.gov/tb/topic/testing/tbtesttypes.htm (2022). Accessed 8 May 2022.

[19] Cattamanchi A (2011). Interferon-gamma release assays for the diagnosis of latent tuberculosis infection in HIV-infected individuals–a systematic review and meta-analysis. J. Acquir. Immune Defic. Syndr..

[20] Lewinsohn DM (2017). Official American Thoracic Society/Infectious Diseases Society of America/Centers for disease control and prevention clinical practice guidelines: Diagnosis of tuberculosis in adults and children. Clin. Infect. Dis..

[21] Neumann PJ, Sanders GD, Russell LB, Siegel JE, Ganiats TG (2016). Cost-Effectiveness in Health and Medicine.

[22] Drummond MF, Sculpher MJ, Claxton K, Stoddart GL, Torrance GW (2015). Methods for the Economic Evaluation of Health Care Programmes.

[23] Xie F (2013). Model-based economic evaluation for medical decision making: Learn from the past and prepare for the future. J. Thorac. Dis..

[24] Weinstein MC (2003). Principles of good practice for decision analytic modeling in health-care evaluation: Report of the ISPOR task force on good research practices—Modeling studies. Value health.

[25] Kerani RP (2020). The epidemiology of HIV among people born outside the United States, 2010–2017. Public Health Rep..

[26] Belknap R (2017). Self-administered versus directly observed once-weekly isoniazid and rifapentine treatment of latent tuberculosis infection: A randomized trial. Ann. Intern. Med..

[27] Linas BP, Wong AY, Freedberg KA, Horsburgh CR (2011). Priorities for screening and treatment of latent tuberculosis infection in the United States. Am. J. Respir. Crit. Care Med..

[28] Sterling TR (2011). Three months of rifapentine and isoniazid for latent tuberculosis infection. N. Engl. J. Med..

[29] Shea KM, Kammerer JS, Winston CA, Navin TR, Horsburgh C, Robert Jr (2014). Estimated rate of reactivation of latent tuberculosis infection in the United States, overall and by population subgroup. Am. J. Epidemiol..

[30] World Health Organization. *Tuberculosis profile: United States of America*. https://worldhealthorg.shinyapps.io/tb_profiles/?_inputs_&entity_type=%22country%22&lan=%22EN%22&iso2=%22US%22 (2021). Accessed 8 May 2022.

[31] Jasmer RM (2004). Recurrent tuberculosis in the United States and Canada: Relapse or reinfection?. Am. J. Respir. Crit. Care Med..

[32] World Health Organization. *Global Health Observatory data repository. Life tables by country_United States*. https://apps.who.int/gho/data/view.main.61780?lang=en (2019). Accessed 8 May 2022.

[33] Balestroni G, Bertolotti G (2012). EuroQol-5D (EQ-5D): An instrument for measuring quality of life. Monaldi Arch. Chest Dis..

[34] Park H-Y, Cheon H-B, Choi SH, Kwon J-W (2021). Health-related quality of life based on EQ-5D utility score in patients with tuberculosis: A systematic review. Front. Pharmacol..

[35] Bauer M (2015). The impact of tuberculosis on health utility: A longitudinal cohort study. Qual. Life Res..

[36] Gold MR, Franks P, McCoy KI, Fryback DG (1998). Toward consistency in cost-utility analyses—Using national measures to create condition-specific values. Med. Care.

[37] Kittikraisak W (2012). Health related quality of life among patients with tuberculosis and HIV in Thailand. PLoS ONE.

[38] Saleem S, Malik AA, Ghulam A, Ahmed J, Hussain H (2018). Health-related quality of life among pulmonary tuberculosis patients in Pakistan. Qual. Life Res..

[39] Center for Medicare and Medicaid Services. *Physician Fee Schedule*. https://www.cms.gov/medicare/physician-fee-schedule/search/overview (2022). Accessed 8 May 2022.

[40] Center for Medicare and Medicaid Services. *Clinical Laboratory Fee Schedule*. https://www.cms.gov/Medicare/Medicare-Fee-for-Service-Payment/ClinicalLabFeeSched/Clinical-Laboratory-Fee-Schedule-Files (2022). Accessed 8 May 2022.

[41] Centers for Disease Control and Prevention. *CDC Estimates for Latent TB Infection (LTBI) Treatment Costs (in 2020 U.S. dollars)*. https://www.cdc.gov/tb/publications/infographic/ltbi-treatment-costs.htm (2021). Accessed 8 May 2022.

[42] Centers for Disease Control and Prevention. *CDC Estimates for TB Treatment Costs (in 2020 U.S. Dollars)*. https://www.cdc.gov/tb/publications/infographic/appendix.htm (2021). Accessed 8 May 2022.

[43] May P (2018). Economics of palliative care for hospitalized adults with serious illness: A meta-analysis. JAMA Intern. Med..

[44] The U.S. Labor Department’s Bureau of Labor Statistics. *US inflation calculator*. www.usinflationcalculator.com (2022). Accessed 8 May 2022.

[45] Briggs A, Sculpher M, Claxton K (2006). Decision Modelling for Health Economic Evaluation.

[46] World Health Organization. *The world health report 2002: reducing risks, promoting healthy life*. https://apps.who.int/iris/bitstream/handle/10665/67454/WHO_WHR_02.1.pdf?sequence=1&isAllowed=y (2002). Accessed 8 May 2022.

[47] Neumann PJ, Cohen JT, Weinstein MC (2014). Updating cost-effectiveness–the curious resilience of the $50,000-per-QALY threshold. N. Engl. J. Med..

[48] Tasillo A (2017). Cost-effectiveness of testing and treatment for latent tuberculosis infection in residents born outside the United States with and without medical comorbidities in a simulation model. JAMA Intern. Med..

[49] Auguste PE, Mistry H, McCarthy ND, Sutcliffe PA, Clarke AE (2022). Cost-effectiveness of testing for latent tuberculosis infection in people with HIV. AIDS.

[50] Cameron D, Ubels J, Norström F (2018). On what basis are medical cost-effectiveness thresholds set? Clashing opinions and an absence of data: A systematic review. Glob. Health Action.

[51] Grosse SD (2008). Assessing cost-effectiveness in healthcare: History of the $50,000 per QALY threshold. Expert Rev. Pharmacoecon. Outcomes Res..

