# Supplementary material for: Cost effectiveness analysis of single and sequential testing strategies for tuberculosis infection in adults living with HIV in the United States
Source: Sci Rep. 2022 Nov 1;12:18349. doi: 10.1038/s41598-022-22721-z (PMC9626471; doi:10.1038/s41598-022-22721-z)
Supplement: Supplementary file 1 — Supplementary Information. [file 41598_2022_22721_MOESM1_ESM.docx]

**Table S1.** Probabilistic distributions and parameters used in the probabilistic sensitivity analysis

| **Parameters** | **Distribution** | |  | |  | |
| --- | --- | --- | --- | --- | --- | --- |
| ***Clinical inputs*** |  |  | **Alpha parameter** | | **Beta Parameter** | |
| HIV prevalence in foreign-born patients | Beta | | 62,170 | | 266,147 | |
| TBI in foreign-born HIV-infected patients | Beta | | 44 | | 96 | |
| TBI in US-born HIV-infected patients | Beta | | 51 | | 1,175 | |
| Sensitivity (foreign-born) |  | |  | |  | |
| TST | Beta | | 97 | | 43 | |
| T-SPOT.TB | Beta | | 82 | | 58 | |
| QFT-Plus | Beta | | 100 | | 40 | |
| Sensitivity (US-born) |  | |  | |  | |
| TST | Beta | | 662 | | 564 | |
| T-SPOT.TB | Beta | | 674 | | 552 | |
| QFT-Plus | Beta | | 828 | | 398 | |
| Specificity (foreign-born) |  | |  | |  | |
| TST | Beta | | 124 | | 16 | |
| T-SPOT.TB | Beta | | 136 | | 4 | |
| QFT-Plus | Beta | | 130 | | 10 | |
| Specificity (US-born) |  | |  | |  | |
| TST | Beta | | 1,183 | | 43 | |
| T-SPOT.TB | Beta | | 1,217 | | 9 | |
| QFT-Plus | Beta | | 1,174 | | 52 | |
| Treatment completion rate of TBI treatment | Beta | | 223 | | 38 | |
| TB risk reduction rate after completed TBI treatment | Beta | | 90 | | 10 | |
| The annual rate of TB reactivation in HIV-infected patients | Beta | | 2,198 | | 118,902 | |
| Treatment success rate of HIV-positive TB cases | Beta | | 266 | | 144 | |
| Mortality rate among not successfully treated TB-HIV patients | Beta | | 68 | | 46 | |
| Annual TB recurrence rate in HIV-infected patients | Beta | | 15 | | 352.12 | |
|  | | | | | | |
| ***Utility inputs*** |  | **Minimum** | | **Likeliest** | | **Maximum** |
| TB disease | Uniform | 0.57 | | - | | 0.77 |
| TB treatment success | Uniform | 0.77 | | - | | 1.00 |
| HIV patient age (years) |  |  | |  | |  |
| Foreign-born | Triangular | 37.6 | | 46.4 | | 56 |
| US-born | Triangular | 42.6 | | 49.6 | | 54.8 |
|  | | | | | | |
| ***Cost inputs* (USD)** |  | | **Mean** | | **Standard error** | |
| Cost per test |  | |  | |  | |
| TST | Gamma | | 35 | | 35 | |
| T-SPOT.TB | Gamma | | 100 | | 100 | |
| QFT-Plus | Gamma | | 62 | | 62 | |
| Cost per case |  | |  | |  | |
| TBI | Gamma | | 710 | | 710 | |
| TB disease | Gamma | | 21,955 | | 21,955 | |
| Palliative care | Gamma | | 27,158 | | 27,158 | |
| TB-related mortality | Gamma | | 37,499 | | 37,499 | |

HIV: human immunodeficiency virus; TBI: tuberculosis infection; PSA: probabilistic sensitivity analysis; QFT-Plus: QuantiFERON-TB Gold Plus; T-SPOT.TB: T-cell spot of the TB assay; TST: tuberculin skin test; TB: tuberculosis

**Table S2.** Base-case results of all testing strategies

| Testing strategy | Total direct cost (USD) | Incremental cost (USD) | QALYs | QALY gained | ICER (USD/  QALY) | Dominated strategy |
| --- | --- | --- | --- | --- | --- | --- |
| TST | 3,344 | - | 0.1014 | - | - | No |
| QFT-Plus | 3,351 | 7 | 0.1051 | 0.0037 | 1,892 | No |
| Confirm positive TST followed by QFT-Plus | 3,353 | 2 | 0.0896 | -0.0156 | -126 | Yes |
| Confirm positive TST followed by T-SPOT.TB | 3,368 | 17 | 0.0847 | -0.0205 | -838 | Yes |
| Confirm negative TST followed by QFT-Plus | 3,377 | 26 | 0.1170 | 0.0119 | 2,185 | No |
| Confirm positive QFT-Plus followed by TST | 3,377 | 1 | 0.0896 | -0.0274 | -33 | Yes |
| No testing | 3,379 | 2 | 0.0618 | -0.0552 | -37 | Yes |
| Confirm negative QFT-Plus followed by TST | 3,379 | 3 | 0.1170 | 0.0000 | 0 | Yes |
| T-SPOT.TB | 3,388 | 11 | 0.0975 | -0.0195 | -570 | Yes |
| Confirm negative TST followed by T-SPOT.TB | 3,398 | 21 | 0.1142 | -0.0028 | -7,737 | Yes |
| Confirm negative T-SPOT.TB followed by TST | 3,406 | 29 | 0.1142 | -0.0028 | -10,616 | Yes |
| Confirm positive T-SPOT.TB followed by TST | 3,425 | 49 | 0.0847 | -0.0323 | -1,506 | Yes |

QALY: quality-adjusted life years; ICER: incremental cost-effectiveness ratio; TBI: tuberculosis infection; QFT-Plus: QuantiFERON-TB Gold Plus; T-SPOT.TB: T-cell spot of the TB assay; TST: tuberculin skin test

Dominated strategy: a strategy with lower QALYs at a higher cost, or lower QALYs at higher ICER than another strategy; dominated strategies are eliminated from further cost-effectiveness analysis


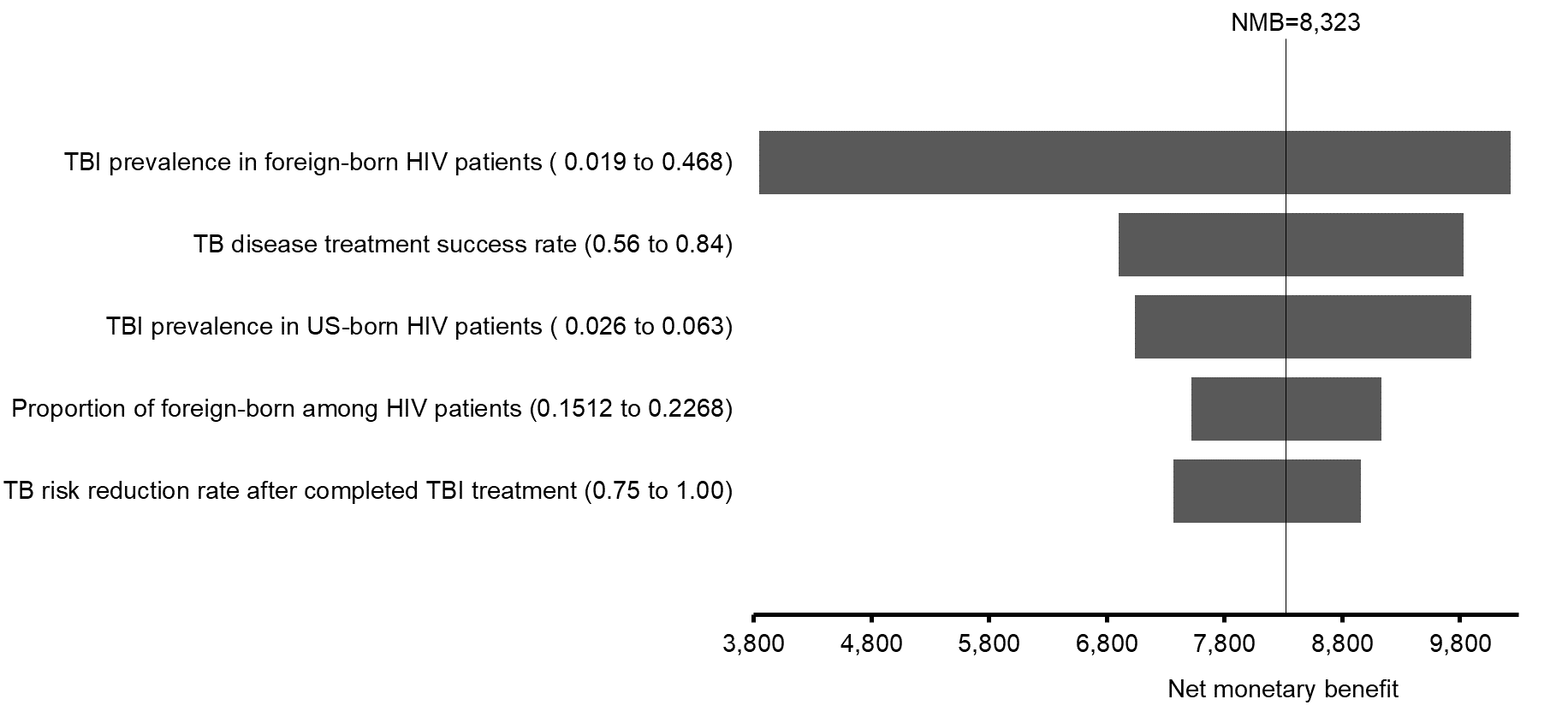


**Figure S1.** Tornado diagram of influential parameters on the preferred cost-effective strategy identified in one-way sensitivity analysis: NMB: Net monetary benefit; QFT-Plus: QuantiFERON-TB Gold Plus; T-SPOT.TB: T-cell spot of the TB assay; TST: tuberculin skin test

The Net Monetary Benefit (NMB) calculation combines cost, effectiveness and willingness-to-pay (WTP) into a single measurement. The strategy with the highest NMB is the preferred cost-effective option. The NMB of a strategy is calculated using the following formula:

NMB=E*WTP-C

where E represents effectiveness, C represents cost, and WTP is the willingness-to-pay.

The NMB of “confirm negative TST followed by QFT-Plus” (the preferred cost-effective strategy in the present model) = (0.1170*100,000)-3,377=8,323

where E=0.1170 QALYs, C=USD 3,377, and WTP adopted 100,000 USD/QALY
